# Zebrafish as a model to investigate a biallelic gain-of-function variant in *MSGN1,* associated with a novel skeletal dysplasia syndrome

**DOI:** 10.1186/s40246-024-00593-w

**Published:** 2024-03-06

**Authors:** Asuman Koparir, Caroline Lekszas, Kemal Keseroglu, Thalia Rose, Lena Rappl, Aboulfazl Rad, Reza Maroofian, Nakul Narendran, Atefeh Hasanzadeh, Ehsan Ghayoor Karimiani, Felix Boschann, Uwe Kornak, Eva Klopocki, Ertuğrul M. Özbudak, Barbara Vona, Thomas Haaf, Daniel Liedtke

**Affiliations:** 1 Institute of Human Genetics, Julius-Maximilians-Universität Würzburg, Biozentrum, Am Hubland, 97074 Würzburg, Germany; 2 Division of Developmental Biology, Cincinnati Children’s Hospital Medical Center, Cincinnati, OH USA; 3 Cellular and Molecular Research Centre, Sabzevar University of Medical Sciences, Sabzevar, Iran; 4 Department of Neuromuscular Disorders, UCL Queen Square Institute of Neurology, London, WC1N 3BG UK; 5 University of Cincinnati College of Medicine, Cincinnati, OH USA; 6 Cellular and Molecular Research Centre, Sabzevar University of Medical Sciences, Sabzevar, 009851 Iran; 7 Department of Medical Genetics, Next Generation Genetic Polyclinic, Mashhad, Iran; 8 Institute for Medical Genetics and Human Genetics, Charité - Universitätsmedizin Berlin, corporate member of Freie Universität Berlin and Humboldt-Universität Zu Berlin, Berlin, Germany; 9 Berlin Institute of Health at Charité - Universitätsmedizin Berlin, Berlin, Germany; 10 Institute of Human Genetics, University Medical Center Göttingen, Göttingen, Germany; 11 Department of Pediatrics, University of Cincinnati College of Medicine, Cincinnati, OH USA; 12 Institute for Auditory Neuroscience and InnerEarLab, University Medical Center Göttingen, Göttingen, Germany

## Abstract

**Background/Objectives:**

Rare genetic disorders causing specific congenital developmental abnormalities often manifest in single families. Investigation of disease-causing molecular features are most times lacking, although these investigations may open novel therapeutic options for patients. In this study, we aimed to identify the genetic cause in an Iranian patient with severe skeletal dysplasia and to model its molecular function in zebrafish embryos.

**Results:**

The proband displays short stature and multiple skeletal abnormalities, including mesomelic dysplasia of the arms with complete humero-radio-ulna synostosis, arched clavicles, pelvic dysplasia, short and thin fibulae, proportionally short vertebrae, hyperlordosis and mild kyphosis. Exome sequencing of the patient revealed a novel homozygous c.374G > T, p.(Arg125Leu) missense variant in *MSGN1* (NM_001105569). MSGN1, a basic-Helix–Loop–Helix transcription factor, plays a crucial role in formation of presomitic mesoderm progenitor cells/mesodermal stem cells during early developmental processes in vertebrates. Initial in vitro experiments show protein stability and correct intracellular localization of the novel variant in the nucleus and imply retained transcription factor function. To test the pathogenicity of the detected variant, we overexpressed wild-type and mutant *msgn1* mRNA in zebrafish embryos and analyzed *tbxta* (*T/brachyury/ntl*). Overexpression of wild-type or mutant *msgn1* mRNA significantly reduces *tbxta* expression in the tailbud compared to control embryos. Mutant *msgn1* mRNA injected embryos depict a more severe effect, implying a gain-of-function mechanism. In vivo analysis on embryonic development was performed by clonal *msgn1* overexpression in zebrafish embryos further demonstrated altered cell compartments in the presomitic mesoderm, notochord and pectoral fin buds. Detection of ectopic *tbx6* and *bmp2* expression in these embryos hint to affected downstream signals due to Msgn1 gain-of-function.

**Conclusion:**

In contrast to loss-of-function effects described in animal knockdown models, gain-of-function of MSGN1 explains the only mildly affected axial skeleton of the proband and rather normal vertebrae. In this context we observed notochord bending and potentially disruption of pectoral fin buds/upper extremity after overexpression of *msgn1* in zebrafish embryos. The latter might result from Msgn1 function on mesenchymal stem cells or on chondrogenesis in these regions. In addition, we detected ectopic *tbx6* and *bmp2a* expression after gain of Msgn1 function in zebrafish, which are interconnected to short stature, congenital scoliosis, limb shortening and prominent skeletal malformations in patients. Our findings highlight a rare, so far undescribed skeletal dysplasia syndrome associated with a gain-of-function mutation in *MSGN1* and hint to its molecular downstream effectors.

**Supplementary Information:**

The online version contains supplementary material available at 10.1186/s40246-024-00593-w.

## Introduction

Skeletal dysplasias are a highly genetically heterogeneous group of skeletal and cartilaginous tissue disorders. The current classification comprises 461 distinct disorders and 437 underlying genes [25, 41]. Because of genetic and phenotypic heterogeneity of skeletal dysplasias, it is often difficult to get an unambiguous clinical diagnosis without molecular analyses. Next generation sequencing (NGS) technologies allow elucidation of the genetic basis of skeletal dysplasias, enabling precise diagnostics and adapted treatments. This method can identify new genetic variants in small patient cohorts or even a single affected individual. However, many of these variants are of uncertain significance/unknown function.

Mesogenin1 (*MSGN1*, OMIM: *612209), a basic-Helix–Loop–Helix transcription factor, is expressed in the presomitic mesoderm (PSM) only at early stages of vertebrate embryogenesis and plays a crucial role in formation of mesodermal progenitor cells during somitogenesis [47]. *Msgn1* null mouse embryos have been shown to be defective in somitogenesis and exhibit somite segmentation defects with absence of all structures arising from the paraxial mesoderm (e.g. musculoskeletal trunk, trunk skeleton, parts of the skin and trunk vasculature). In addition, these mouse embryos displayed prominent tail segmentation defects. Reduced expression of components of the Delta-Notch signaling pathway and the segmentation clock oscillator was observed within the PSM [47]. In addition to the loss-of-function phenotype, ectopic expression of *Msgn1* in mice suppresses notochord cell differentiation and promotes a PSM stem-cell fate [2]. The notochord is a primary structural element, a source of patterning signals, and is thereby essential for locomotion by enabling early cartilage and vertebrae development during vertebrate embryogenesis [6, 37]. In accordance with mouse experiments, zebrafish embryos injected with *msgn1* mRNA exhibited severe truncation of tail structure, e.g. loss of tailbud and notochord cells [46]. This altered development is induced by disturbed expression of essential PSM regulators like *tbxta* (alias: *TBXT* (human), *T/brachyury/bra* (mouse), *no tail/ntla/zft/ta* (zebrafish)), which were significantly reduced in the posterior tailbud and in the axial region after gain of Msgn1 function [46]. The correct function of the PSM during early development is the prerequisite to subsequent processes like somite formation, notochord development and the segmentation clock itself. Its function involves a highly sophisticated network of different molecular factors in addition to MSGN1. These comprise WNT, FGF, retinoic acid, Delta-Notch signaling pathways and T-box transcription factors [23, 35] and are evolutionarily conserved among vertebrate species. MSGN1 functions as a transcription factor, which is regulated by these factors, but moreover orchestrates correct transcription of several essential effectors within this network [2].

Here, we have investigated the molecular pathogenesis of a novel form of skeletal dysplasia in the offspring of consanguineous Iranian parents and identified a novel homozygous c.374G > T, p.(Arg125Leu) variant in *MSGN1* (NM_001105569) in the affected child. To test the pathogenicity of this variant, we overexpressed either wild-type or mutant *msgn1* in zebrafish via mRNA injection and analyzed *tbxta* expression in the tailbud. Consequences of enhanced Msgn1 activity on early development were further analyzed in vivo in zebrafish embryos. Mosaic transient-transgenic overexpression of *msgn1* showed spatial and temporal restricted consequences on tail development by influencing Delta-Notch active cells in the posterior progenitor zone and by inducing ectopic gene expression.

## Results

### Clinical description

**Fig. 1:**
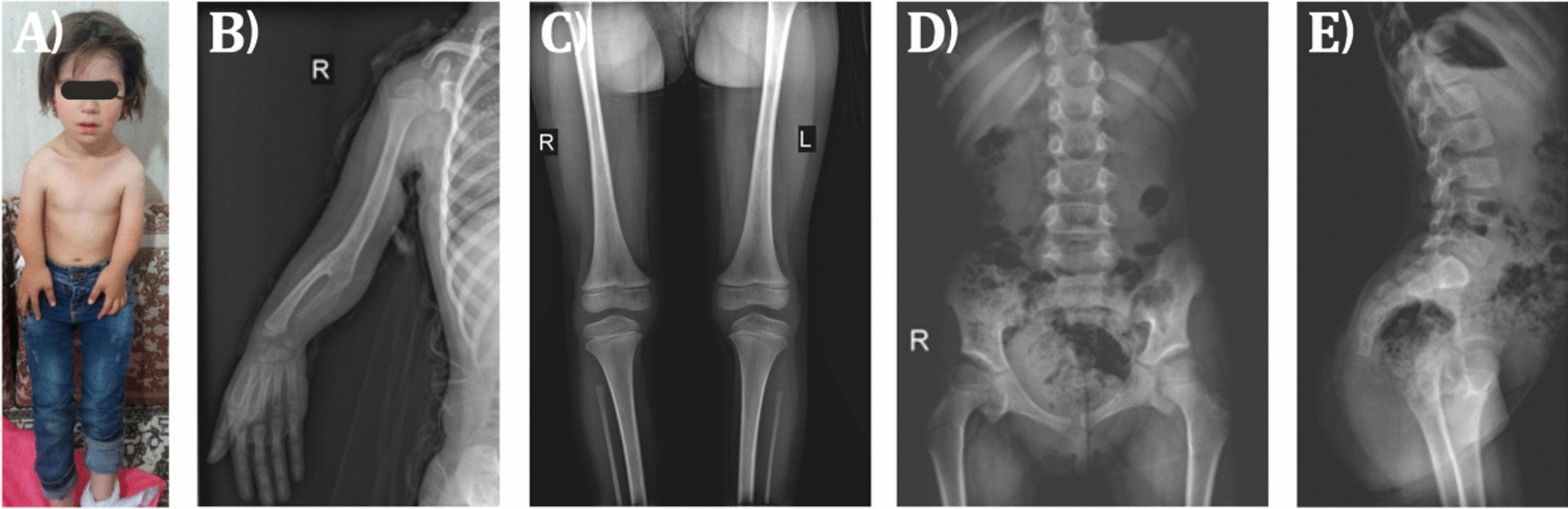
**A** Short stature and mesomelic shortening of arms in the patient. **B**–**E** Skeletal X-ray surveys of the patient's right upper extremity (**B**), lower extremities (**C**), anterior view of the lower spine and pelvis (**D**), and lateral view of the lower spine and pelvis (**E**)

The patient was the first child born to a gravida 2, para 1 mother. The healthy parents were second-degree cousins. On physical examination, the female proband had mild facial dysmorphism including hypertelorism, epicanthus, midface hypoplasia, mild retrognathia, and short stature with noticeable mesomelic shortening of the arms. She also presented dysplastic neck-thorax region with a prominent trapezoid muscle and arched clavicles (Fig. 1A). Radiographic images revealed humero-radial and radio-ulnar synostosis. The radii were severely shortened and hypoplastic, the ulnae were rudimentary with only their proximal part present. The scaphoid bone was only present as a tiny nucleus on the left side, while it was ossified on the right side (Fig. 1B). The diaphysis of the femora was very thin. The fibulae were clearly shortened and too thin (Fig. 1C). The iliac bones were somewhat narrow, the ramus inferior of the pubic bone was missing, and the ischium was only rudimentary (Fig. 1D). In addition, there was a hyperlordosis and mild scoliosis of the spine (Fig. 1E).

### Whole exome sequencing revealed a likely disease-causing variant in *MSGN1*

**Fig. 2:**
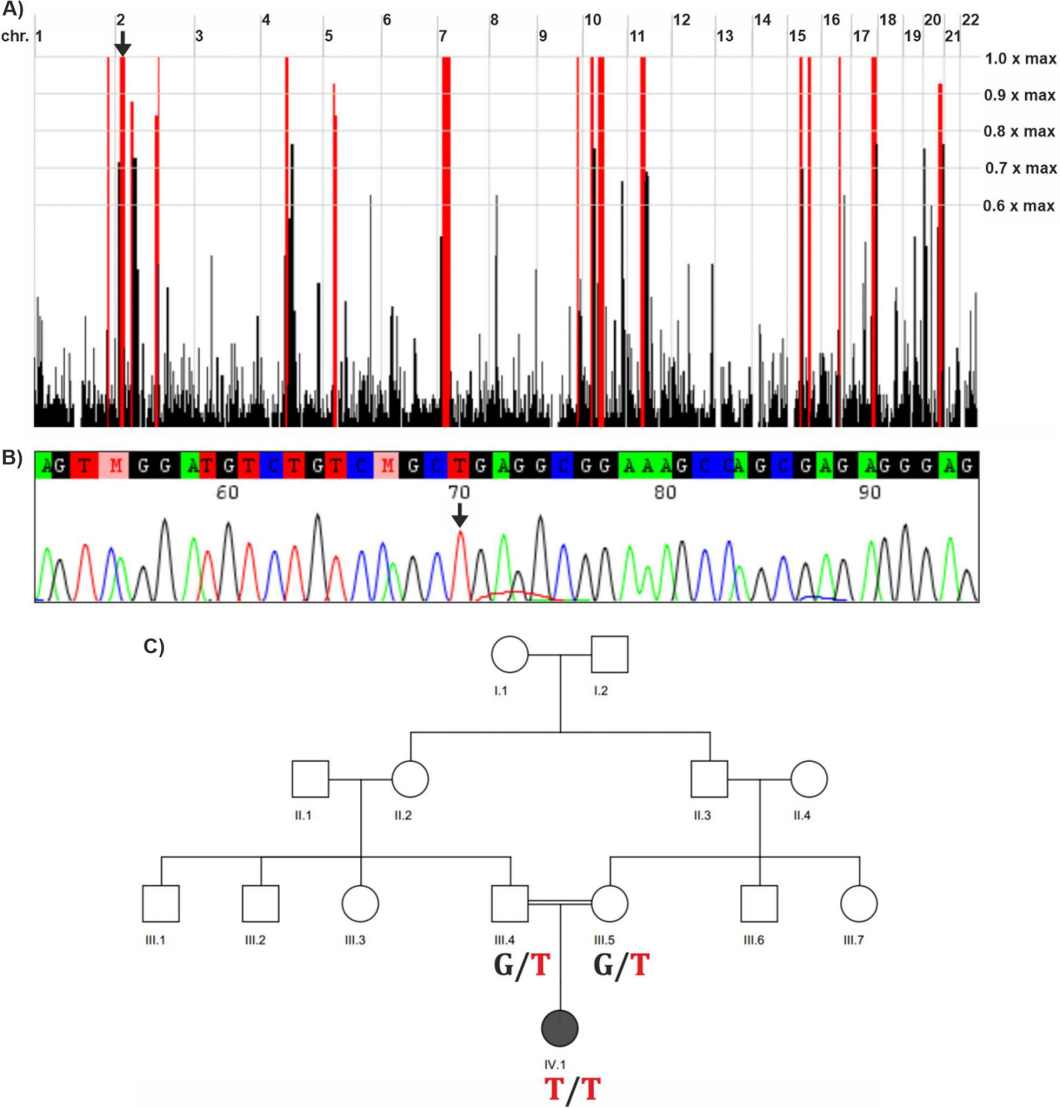
Genetic results of the family. **A** Homozygous intervals in the patient’s genome, as determined by HomozygosityMapper. *MSGN1* resides in a ~ 13.2 Mb homozygous interval on chromosome 2 (black arrow). **B** Sanger sequencing electropherogram of the patient’s variant in *MSGN1*. The mutated base is indicated by a black arrow. **C** Segregation of the *MSGN1* variant within the family

Whole exome sequencing (WES) analysis detected a rare homozygous missense variant in *MSGN1* (NM_001105569.3: c.374G > T, p.(Arg125Leu); NC_000002.11:g.17998159G > T). This variant is listed 11 times in heterozygous but not in homozygous state in the population database gnomAD v.4.0.0. The variant affects a conserved amino acid residue within a highly conserved bHLH domain in MSGN1 (Additional file 2: Fig. S1 and Additional file 3: Fig. S2). Different *in-silico* prediction tools such as CADD (Combined Annotation Dependent Depletion; [15, 30]), REVEL [11] and AlphaMissense [5] classified this variant as pathogenic (Additional file 8: Table S1). Moreover, the variant was predicted by SpliceAI to have no splicing effect [12]. Furthermore, we visualized the theoretical three-dimensional (3D) representation of both wild-type and mutant forms of the MSGN1 protein, to see any alteration in the structure and conformation of the protein (Additional file 2: Fig. S1A, B). The affected Arginine 125 within the MSGN1 protein is located at the second position of the evolutionary highly conserved basic helix-loop-helix (bHLH) protein domain at the beginning of the larger, first alpha-helix. The exchange from Arginine (negative hydropathy index, amphipathic and polar, positive charged side chain) to Leucine (positive hydropathy index, aliphatic and nonpolar, no charge) is biochemically profound. However, the exchange is predicted to not abrogate the helix structure but to cause misfolding of the encoded protein at the transition between the alpha-helix and the N-terminus (Additional file 2: Fig. S1A, B). According to HomozygosityMapper [34], the detected *MSGN1* variant lies within an approximately 13.2 Mb homozygous interval on chromosome 2 (Fig. 2A). Sanger sequencing analysis confirmed WES findings and demonstrated that both parents are heterozygous carriers for the variant (Fig. 2B, C). An additional WGS analysis did not detect other candidate variants with higher pathogenic potential in known skeletal dysplasia genes and surrounding non-coding sequences.

### In vitro analysis of intracellular MSGN1 localization

**Fig. 3:**
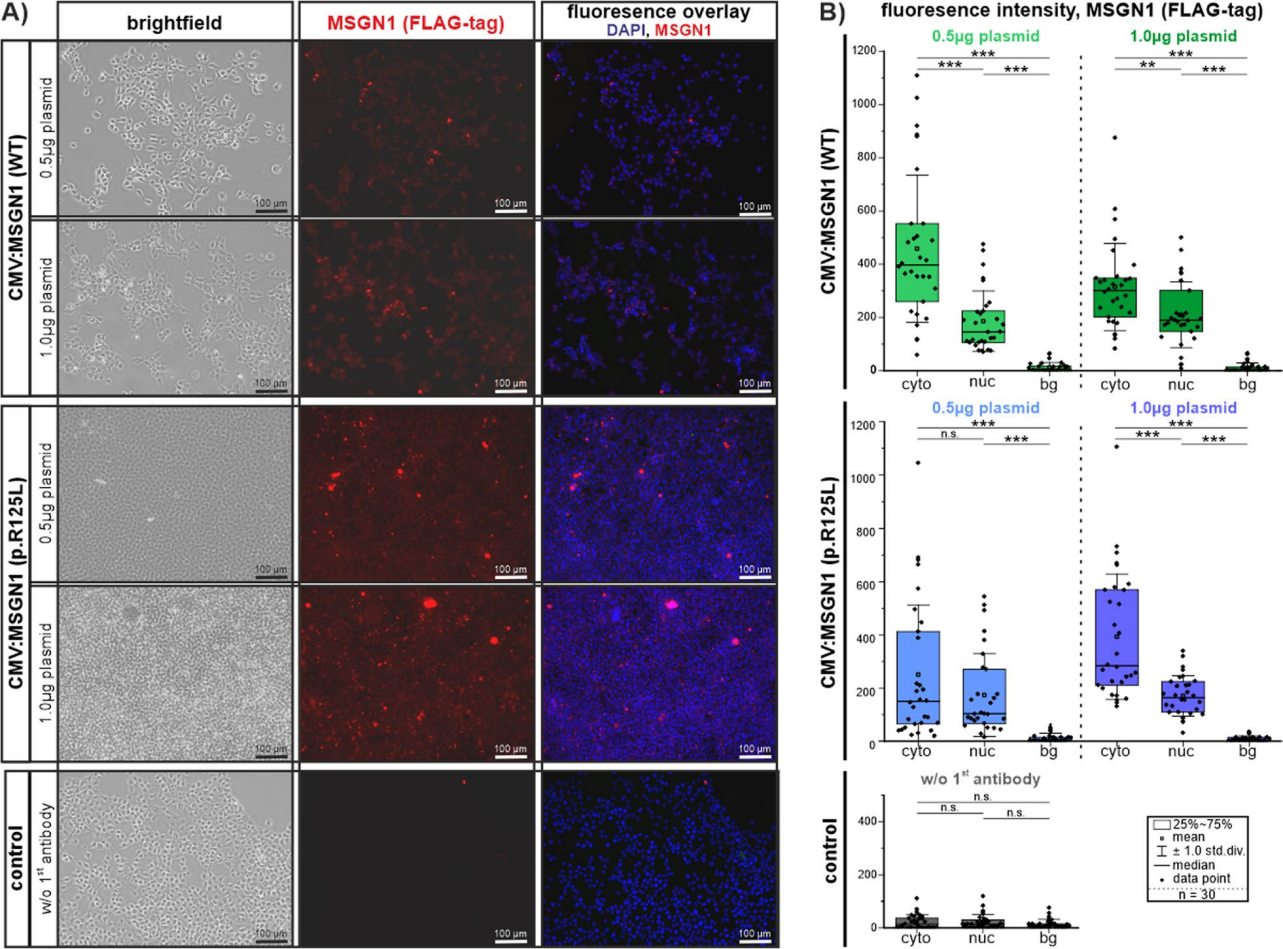
In vitro transfection of HEK 293T cells with CMV:MSGN1 (WT)-FLAG-tag and CMV:MSGN1 p.(Arg125Leu)-FLAG-tag plasmids at different concentrations. **A** Immunofluorescence showing expression of tagged MSGN1 proteins (WT) or p.(Arg125Leu) after 48h of transfection. Corresponding experimental controls are shown in Additional file 4: Fig. S3A. **B** Quantification of MSGN1 localization in transfected cells. Fluorescence signals in a by single z-plane were visualized by confocal laser-scanning microscopy and subsequently signal intensity was measured in single cells (cyto: cytoplasm; nuc: nucleus) and outside of cells (bg: background). Graphs show signal intensity measurements of the RFP channel (MSGN1 Flag-Tag) of 30 cells per experimental group and 30 background positions. Signal intensity measurements of the corresponding DAPI channel for nucleus identification are given in Additional file 4: Fig S3B. Values are given in Additional file 1: Excel file S1

At first, we tested whether the newly identified MSGN1 variant and its predicted misfolding results in retention of MSGN1 protein in the cellular cytoplasm and nuclear exclusion, consistent with a loss-of-function of the transcription factor. HEK 293T cells were transfected with CMV-promoter driven expression plasmids at different concentrations incorporating either wild-type or c.374G > T, p.(Arg125Leu) MSGN1-FLAG-tag variants. Subsequent protein visualization was performed after 48 h via immunofluorescence by utilizing the protein tagged (Myc-DDK/FLAG). Transfected HEK 293T cells showed expression of both MSGN1 variants at different plasmid concentrations and did not imply loss of MSGN1 protein or restrained protein localization (Fig. 3A; controls Additional file 4: Fig. S3A).

To further quantify intracellular localization of the MSGN1 proteins in the cytoplasm or in the nucleus of transfected cells we subsequently quantified fluorescence signal intensities of MSGN1-FLAG-tag and DAPI by confocal laser-scanning microscopy (LSM; Fig. 3B and Additional file 4: Fig. S3B). Measurement of MSGN1 fluorescence signal intensity did not reveal any difference in localization between MSGN1 (WT)-FLAG-tag or MSGN1 (Arg127Leu)-FLAG-tag variants.

### Gain of *msgn1* activity results in developmental defects during early stages of zebrafish development

**Fig. 4:**
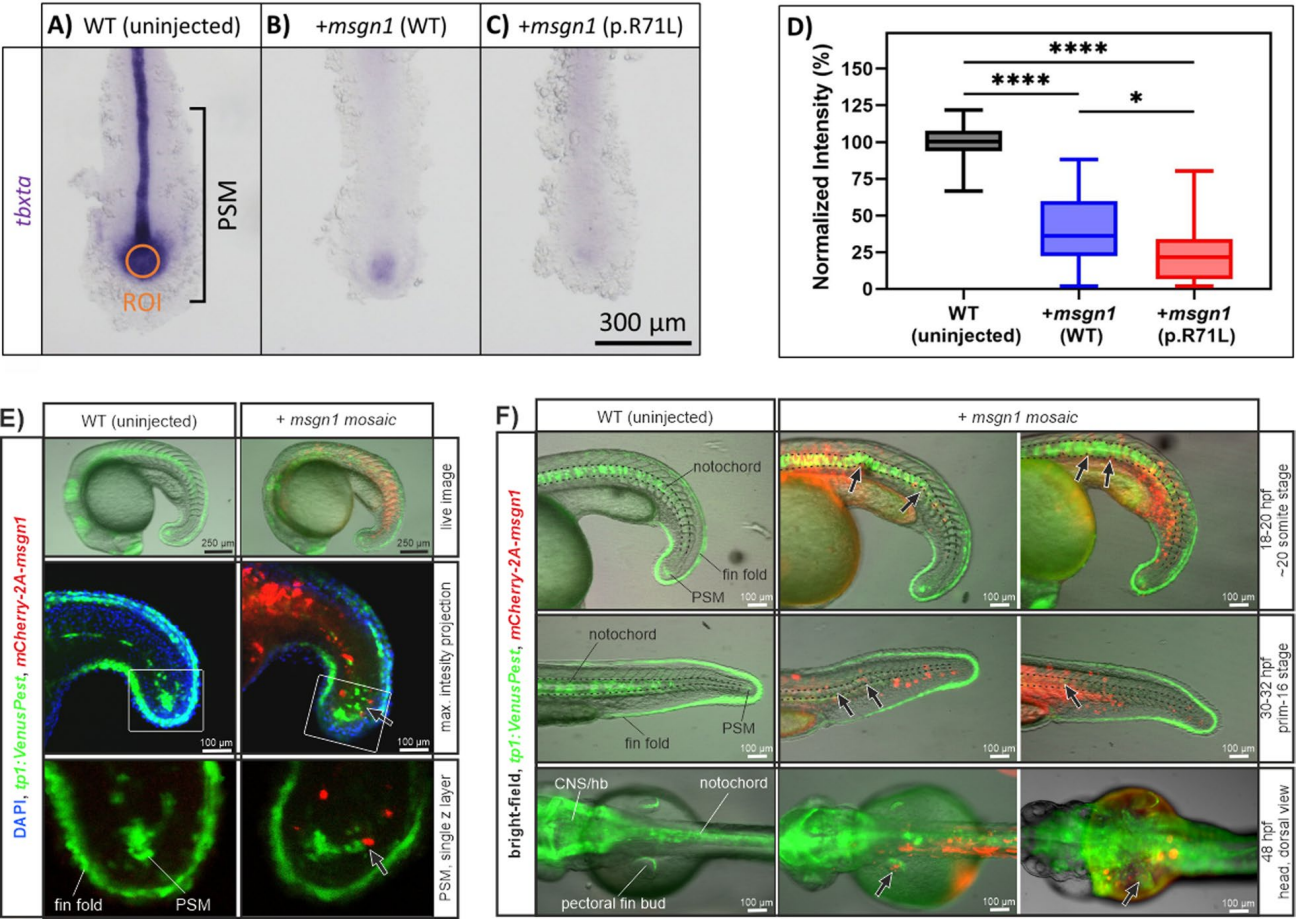
In situ hybridization images of *tbxta* (*no tail,* *Brachyury*) expression in uninjected wild-type controls (**A**), wild-type *msgn1* RNA (**B**) and mutant *msgn1* RNA p.(Arg71Leu) in zebrafish (≈ p.(Arg125Leu) in humans) injected embryos (**C**). **D** Quantification of *tbxta* ISH signal in posterior PSM cells (orange ROI mark) is shown by normalized intensity comparison between the three groups of three independent experiments (WT (uninjected): 49 embryos; + *msgn1* (WT) RNA: 58 embryos; + *msgn1* p.(Arg71Leu) RNA: 54 embryos). Mosaic transient-transgenic overexpression of wild-type *msgn1* results in partial disruption of Notch signals in PSM cells (**E**) and influences normal notochord (marked with dashed lines) and pectoral fin bud development (marked with arrow). **F** In vivo images show representative embryos of three independent injections experiments (overall: 37 imaged embryos; WT: 5 embryos; + *msgn1* mosaic: 32 embryos). ISH signal quantification values and injection statistics are given in Additional file 1: Excel file S1

Next we investigated the possible pathogenic effects of the variant on Mesogenin1 activity in zebrafish a in vivo model. To this end, we overexpressed either wild-type or the variant version (p.(Arg71Leu) in zebrafish corresponding to p.(Arg125Leu) in humans) of Msgn1 via mRNA injection at one-cell stage. We examined *tbxta* expression, which is regulated by Msgn1 [4, 46], in the tailbud at 8-somite stage (Fig. 4A–C). We found that overexpression of both wild-type and *msgn1* p.(Arg71Leu) mRNA significantly reduced *tbxta* expression in the tailbud compared to uninjected wild-type embryos (*P* < 0.0001). In addition, we also observed a more severe effect by *msgn1* p.(Arg71Leu), compared to *msgn1* wild-type mRNA (*P* = 0.0186), suggesting that the variant leads to gain-of-function in Mesogenin1 activity (Fig. 4D).

To investigate additional consequences of enhanced Msgn1 activity on embryonic development we utilized a mosaic transient-transgenic overexpression method. In short, microinjection of a DNA plasmid containing a *mCherry-2A-msgn1* coding sequence (driven by the zebrafish *msgn1* promoter) into single cells of early blastula stage zebrafish embryos drives clonal *msgn1* expression during development. Ectopic *msgn1* expressing cells are marked by mCherry fluorescence. By application of this method in the *tp1:VenusPEST* transgenic zebrafish background, we were able to investigate *msgn1* overexpressing cells and Notch signaling during development simultaneously by in vivo fluorescence visualization. We observed that mCherry positive/*msgn1* expressing cells disturb Notch positive cells within the PSM (18–20 hpf; Fig. 4E). The cell population resembles *tbxta* expressing posterior PSM cells at the maturation zone, giving rise to notochord and somite tissues [8, 17]. Other Notch positive tissues, e.g. dorsal fin fold cells, were not influenced by *msgn1* implying PSM specific effects. PSM disruption is associated with developmental defects whose severity is linked to the amount of *msgn1* overexpressing cells in the PSM region (Additional file 5: Fig S4). Investigation of these embryos at later development stages further showed that mosaic embryos displayed altered notochord and pectoral fin structures (e.g. wavy notochord outgrowth, disruption of Notch signals in notochord cells and in pectoral fin buds, marked by arrows in Fig. 4F).

**Fig. 5:**
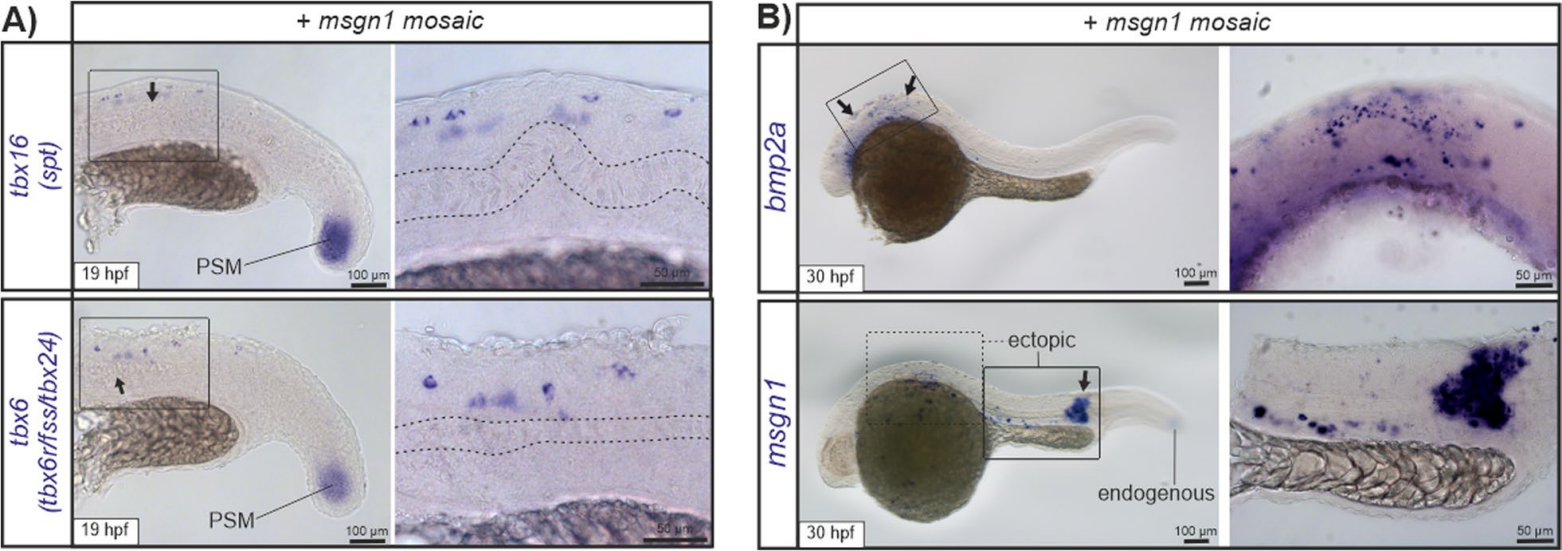
**A** I*n situ* hybridization images of *tbx6* (*tbx6r/fss/fussed somites/tbx24*) and *tbx16* (*spt/spatetail*) expression in injected embryos 19 hpf showing mosaic transient-transgenic overexpression of wild-type *msgn1.* Expression of *tbx6* and *tbx16* was detected in PSM cells (endogenous expression) and within the trunk, colocalizing with notochord alterations (shown in higher magnification). **B** In 30 hpf old *msgn1* mosaic embryos, ectopic expression of *bmp2a* was detected in the head region, consistent with strong ectopic *msgn1* expression in trunk and head regions of injected embryos at this stage

To further decipher potential downstream effects of ectopic Msgn1 activity, we investigated expression of previously described MSGN1 downstream target genes in mosaic zebrafish embryos [2]. Visualization of *tbx6* and *tbx16* via in situ hybridization showed that both factors are endogenously expressed within the PSM and are additionally expressed within the trunk at regions depicting notochord alterations (19 hpf; Fig. 5A). Moreover, strong ectopic expression of *bmp2a* and *msgn1* was detected within the head and trunk regions of 30 hpf embryos (short staining time with low levels of endogenous ISH transcript signal visible; Fig. 5B). Detection of *msgn1* expression via in situ hybridization was in accordance with *mCherry-2a-msgn1* fluorescence expression patterns (see Figs. 3F and 5E) and probably detects primarily transgene induced *msgn1* expression in these mosaic embryos.

General overexpression either of human *MSGN1* (WT) or *MSGN1* p.(Arg125Leu) variants in zebrafish embryos resulted in similar developmental consequences during early developmental stages as gain of zebrafish *msgn1* in mosaic embryos, e.g. notochord bending and disruption of PSM cells (used plasmids: CMV:MSGN1-Flag-tag (WT) and CMV:MSGN1-Flag-tag p.(Arg125Leu), Additional file 6: Fig. S5 and Additional file 1: Excel file S1). This observation implies retained function of human MSGN1 and of the patient derived MSGN1 variant also in zebrafish. Further tissue-restricted mosaic expression of zebrafish *msgn1* p.(Arg71Leu) and human *MSGN1* p.(Arg125Leu) variants in PSM and trunk cells under *msgn1* promoter control in *tp1:VenusPEST* embryos resulted in similar developmental consequences like *msgn1* wild-type injected embryos, but display more severe manifestations and higher rates of severe phenotypes in injected embryos (msgn1:mCherry-2A-msgn1 vector: 15% (n = 197) and 20% (n = 92); msgn1:mCherry-2A-msgn1 p.(Arg71Leu) vector: 32% (n = 50); msgn1:mCherry-2A-MSGN1 p.(Arg125Leu) vector: 27% (n = 104); Additional file 7: Fig. S6 and Additional file 1: Excel file S1).

## Discussion

In our study, we identified a homozygous missense variant c.374G > T, p.(Arg125Leu) in *MSGN1* via exome sequencing to be potentially associated with prominent skeletal abnormalities in a single patient. *MSGN1* missense variants have not yet been associated with a human Mendelian disease. Previously, eleven *MSGN1* missense variants have been reported in ClinVar, but all lack detailed zygosity and phenotype information or segregation analyses and are all classified as variants of uncertain significance (Tab. S3). Several structural variants (gains and losses) including the *MSGN1* locus are additionally described in ClinVar, but all these alterations affect larger genomic regions incorporating several coding genes and therefore do not allow the conclusion that *MSGN1* is disease causing (Tab. S4). The aim of this study was to provide molecular insights into our patient’s phenotype and to link the observed malformations to known MSGN1 functions during development using zebrafish.

In contrast to loss of function phenotypes in MSGN1 animal models (short tail, prenatal lethal, absent notochord), we did not observe prominent vertebral segmentation or axial skeleton defects in our patient. We therefore propose the detected *MSGN1* variant has a gain of function effect, which could explain only mildly affected vertebrae of the proband. Initial in vitro investigations clarified, that the corresponding protein of the novel MSGN1 p.(Arg125Leu) variant is still able to localize into the nucleus and therefore may act as a transcription factor. Previously published results [46] in zebrafish showed that elevated *msgn1* activity reduces *tbxta* expression in the PSM. Our own experimental data are in accordance with this observation and further demonstrate that the corresponding *msgn1* p.(Arg71Leu) variant results in a stronger gain of function effect hinting to raised Mesogenin1 activity (Fig. 4D and Additional file 7: Fig. S6). TBXT, the prototypical T-box transcription factor, is essential for the formation and differentiation of posterior mesoderm and for axial development in all vertebrates. Homozygous variants in *TBXT* orthologues cause embryonic lethality, while heterozygous variants are associated with short tail mice [16, 33] and zebrafish (*no tail* mutant; [27, 38, 42]). In humans, a homozygous *TBXT* variant (c.512A > G, p.(His171Arg), NM_003181), has been associated with sacral agenesis, abnormal ossification of vertebral bodies and persistent notochord canal [29]. In chicken *T* is expressed in the lateral plate mesoderm at the onset of limb bud formation [22]. T controls *TBX6* expression, which is known to regulate activation of *MSGN1* [45]*. TBX6* variants cause spondylocostal dysostosis in human [36]. However, ectopic *Tbx6* gain-of-function within the segmented paraxial mesoderm and its derivatives in mice are associated with appendicular skeletal defects including hypoplastic scapula, small limbs, shortened and malformed humerus, radius, ulna, femur, tibia and fibula, resembling *Tbx15* null mouse embryos and Cousin syndrome in humans (OMIM #260660) [18, 43]. Since gain of *Tbx6* function in mice results in short stature and severe limb defects [45], we hypothesize that *MSGN1* gain of function is responsible for short stature and limb abnormalities in our patient. The expression of T-box transcription factors like *tbxt*, *tbx6*, *tbx16*, and *tbx16l* in zebrafish is regulated by Msgn1 [4, 13, 24]. Our transient mosaic experiments in zebrafish and previously published zebrafish experiments further show ectopic *tbx6* expression via gain of Msgn1 function (Fig. 4 and [46]). To our knowledge, no skeletal dysplasia has been associated with biallelic pathogenic gain of function variants. However, there are some examples in the literature for biallelic pathogenic gain of function variants, which affect other physiological systems e.g. the immune system (*NLRP1*, *STAT2*) [7, 9].

*MSGN1* plays an important role in notochord maintenance and development, that expresses genes such as *SOX9* which are characteristic of chondrogenesis [37]. Prominent functions of Delta-Notch signals during early posterior tail development have been intensively investigated and are necessary to synchronize PSM cells within the somitogenesis clock of vertebrates [19–21]. We visualized Delta-Notch signalling for in vivo observation of developmental defects in the posterior PSM maturation zone and in the notochord. Our zebrafish results imply that gain of *msgn1* function in single cells of the posterior progenitor zone (maturation zone in the posterior PSM) disturbs the structure of Delta-Notch positive cells which give rise to somite, paraxial mesoderm and notochord tissues during later developmental stage [8, 31]. We subsequently observed notochord bending and potential disruption of pectoral fin buds/upper extremity. The later might result from *msgn1* ectopic function on mesenchymal stem cells or on chondrogenesis in this region [3, 28, 40]. Interestingly within this context, we found that transcriptional *Msgn1* activity results in ectopic *bmp2a* expression (besides action on *tbx6* and *tbxta*). Bmp2 is described as a direct MSGN1 target [2] and is known to be associated with prominent skeletal anomalies in patients, like brachydactyly type A2 (OMIM #112600), as well as short stature, facial dysmorphism, and skeletal anomalies with or without cardiac anomalies (OMIM #112261). Further studies will have to clarify, if the long-term ectopic expression of downstream effectors of MSGN1 is also the driver of long-term developmental alterations in the current patient, since spatio-temporal *MSGN1* expression is usually tightly regulated during development.

Collectively, the studied patient presents a novel *MSGN1*-associated skeletal syndrome in humans, highlighting the importance of the temporally and spatially appropriate MSGN1 activity in various developmental pathways. However, additional patients/families are needed to unequivocally link *MSGN1* gain-of-function to the described syndrome.

## Materials and methods

### Whole exome sequencing and database research

Exome capture was performed according to the Illumina Nextera Rapid Capture Enrichment library preparation protocol (individuals IV.1, III.4 and III.5) using 50 ng of genomic DNA. Paired-end sequencing of the libraries was performed with a NextSeq500 sequencer and the v2 reagent kit (Illumina, San Diego, California, USA). Sequences were mapped to the human genome reference (NCBI build 37/hg19 version) using the Burrows-Wheeler Aligner. Aligned reads ranged between 85,736,827 and 99,719,268. The mean coverage was ≥ 50 with 91.2%. 99% of the exome were covered at least 10x. A total of 678,344–961,783 variants per sample were called and analyzed using GensearchNGS software (PhenoSystems SA, Braine le Chateau, Belgium). Variants with a coverage of ≤ 10, a Phred-scaled quality of ≤ 15, a frequency of ≤ 15, and a MAF of ≥ 2% were neglected. Six control samples from healthy individuals were used for filtering out platform artefacts. Alamut Visual (Interactive Biosoftware, Rouen, France) software including prediction tools like SIFT, MutationTaster, PolyPhen-2, CADD-, and REVEL-Score was used for variant prioritization. Potential effects of a variant on pre-mRNA splicing were evaluated by SpliceAI, SpliceSiteFinder-like, MaxEntScan, NNSPLICE, GeneSplicer, Human Splicing Finder, ESEfinder, RESCUE-ESE, and EX-SKIP. Population databases like gnomAD v4.0.0, and GME revealed whether a variant has been previously found. Protein expression, structure, and functional aspects were investigated with UniProt and The Human Protein Atlas. Information on mouse and zebrafish models was retrieved from the MGI and ZFIN database, respectively.

### Sanger sequencing

*MSGN1* exon one was amplified by a touchdown PCR program using primers in the flanking introns (forward: 5´-GGTGGACTACAATATGTTAGCTTTCC-3´ and reverse: 5´-TAGACAGGTGGCAGGTAATTCC-3´). A clean-up step with ExoSAP-IT (Applied Biosystems, Foster City, USA) was followed by the sequencing reaction using the BigDye Terminator Cycle Sequencing Kit v1.1 (Applied Biosystems, Waltham, USA). Sequencing was conducted on a 3130XL capillary sequencer (Applied Biosystems, Waltham, USA) and data analysis was performed with Gensearch (PhenoSystems SA, Braine le Chateau, Belgium).

### Whole genome sequencing

Short read genome sequencing was performed to exclude structural variants and non-coding variants in known skeletal dysplasia genes. In brief, genomic DNA was isolated from peripheral blood and sequenced at 30 × coverage using the Illumina TruSeq PCR-free protocol at the West German Genome Center (WGGC). Reads were aligned to the human genome build GRCh37/hg19 using BWA-MEM 0.7.17. The VarFish software was used for filtering and interpretation of variants including SVs (caller: Delly v.0.8.1) according to an in-house SOP [10].

### In vitro experiments and intracellular MSGN1 localization measurements

Transfections were performed with 2.5 × 10^5^ HEK 293T cells per well in 12-well plates. HEK 293T cell were initially grown on glass cover slides and subsequently transfected with two different amounts of DNA plasmids containing either the coding sequence of wild-type MSGN1 (WT) or of the MSGN1 p.(Arg125Leu) variant coupled to a FLAG-tag under control of the *CMV* promoter. 0.5 µg and 1.0 µg plasmid DNA per 100 µl total transfection volume was used with 2 to 3 µl FuGene HD transfection agent per well (Promega, Madison, USA, product-nr. E2311). 48 h after transfection, cells were paraformaldehyde fixed and immunofluorescence staining of Flag-tag was performed by standard protocols for cell HEK 293T cell cultures (primary antibody: monoclonal DYKDDDDK Tag Recombinant, Thermo Fisher Scientific, Waltham, USA, product-nr. 701629, RRID: AB_2532497, 1:250 dilution (2.5 µg/ml); secondary antibody: goat anti-rabbit-Alexa 594, Invitrogen/Thermo Fischer Scientific, product-nr. A-11012, RRID: AB_2534079, 1:1000 dilution (1 µg/ml)) and DAPI (Sigma-Aldrich, St. Louis, USA; product-nr. D9542, 1:5000 dilution (4 µg/ml)). Corresponding controls were performed by transfection of an CMV:GFP-Tag plasmid (positive control), by including non-transfected control cells, by omitting primary or secondary antibody incubations (negative controls). Images were taken with a Keyence BZ-X800 fluorescence microscope (Keyence, Osaka, Japan).

Intracellular localization of MSGN1 proteins was quantified by analysis of single z-level immunofluorescence images acquired by laser scanning confocal microscopy (Nikon A1 + , Nikon NIS-Elements software, Nikon Instruments, Tokyo, Japan) via ImageJ/Fiji software ( https://fiji.sc/) [32]. In contrast to analysis by classical fluorescence microscope techniques, usage of LSM enables high resolution optical sectioning of fluorescence signals in a narrow single z-level at height of the nucleus and removes out-of-focus fluorescence signals outside of the focal plane in conjunction with strict pinhole settings. Images were taken at 600-fold magnification, in two channels (MSGN1-tag: 561 nm laser excitation, 610–620 nm emission; DAPI in nucleus: 405 nm laser excitation, 470–480 nm emission), at a resolution of 1024 × 1024 pixel corresponding to a 205 × 205 µm area. To circumvent light scattering, smallest pinhole settings per laser channel were chosen. For each transfection group, three non-overlapping images were acquired, subsequent 10 cells per image were randomly selected and fluorescence intensity values were measured in ImageJ/Fiji software by straight line histograms (line length: 10 µm (50 pixel), line thickness: 2 µm (10 pixels), mean value per pixel position in length were measured at 50 positions per line and channel). Line positions were adjusted to start in the cytoplasm (low DAPI intensity signal) and end in the nucleus (high DAPI intensity signal), with line midpoint corresponding to nucleus border (position #25). Cytoplasmic values were defined as position #15, 2.5 µm away from nucleolus border. Nuclear values were defined as position #35, 2.5 µm away from nucleolus border. Background values were defined as position #15 and #35 values measured outside of cell bodies at five positions per image.

### Zebrafish animal maintenance and lines

Laboratory zebrafish embryos (*Danio rerio*) of the *AB/TU* and *AB/AB* wild-type strain (ZDB-GENO-010924-10; ZDB-GENO-960809-7) and transgenic Tg(*EPV.Tp1-Mmu.Hbb:Venus-Mmu.Odc1*) (ZDB-TGCONSTRCT-120419-4, *tp1:VenusPEST*, [26]) were maintained as previously described under standard aquatic conditions at an average of 24 °C water temperature [1, 44]. Embryos were staged by morphological characteristics according to Kimmel et al. [14]. “hpf” and “dpf” indicate embryonic development in hours/days-post fertilization at 28.5 °C incubation temperature, respectively. All procedures involving experimental animals were performed in compliance with local animal welfare laws, guidelines, and policies. All presented experiments have been performed in zebrafish embryos and larvae younger than 5 dpf, before free-swimming and independent feeding, and thus are not regulated as animal experiments in Germany under current legislation.

### Mosaic transient-transgenic msgn1 overexpression and in vivo visualization

Mosaic transient-transgenic overexpression was performed by microinjection of a previously published plasmid containing a zebrafish *msgn1* promoter fragment driving 2A-coupled expression of *mCherry* fluorescence protein and wild-type zebrafish *msgn1* [46]. Injection of this plasmid results in prominent and severe tail malformations due to gain of Msgn1 function in mesodermal progenitor cells. Injection of *msgn1* injection solution (DNA plasmid was diluted in water to a final concentration of 25 ng/µl and admixed with 0.05% Phenol-red (pH 7.0) for visualization of injection solution) into only one cell of early blastula zebrafish embryos (4-cell stage/1.0 hpf up to 16-cell stage/1.5 hpf) resulted in a fraction of *msgn1* overexpressing cells within developing embryos. In addition to zebrafish wild-type *msgn1* (msgn1:mCherry-2A-msgn1), plasmids containing either a zebrafish *msgn1* p.(Arg72Leu) variant (msgn1:mCherry-2A-msgn1 (p.R71L)), or a human MSGN1 p.(Arg125Leu) variant (msgn1:mCherry-2A-MSGN1 (p.R125L)) were injected in a similar way into zebrafish embryos and investigated.

Injected embryos were in vivo analyzed under a Fluorescence Stereomicroscope (Leica S8 APO equipped with Leica GFP and DSR filter sets (filter nr. 10447408 and 10447412), Leica Miscrosystems, Wetzlar, Germany) at different time points during embryonic and early larval development (approximately between 16 and 48 hpf) for cells showing *msgn1* overexpression by mCherry fluorescence and for resulting developmental consequences. Injection was performed in embryos of a *tp1:VenusPEST* transgenic line [26], which enables in vivo visualization of Notch signaling due to expression of a short-half-life version of the fluorescence protein Venus under the *tp1* promoter element. The *tp1* element contains 12 EBV terminal protein 1 (TP1) gene promoter fragments for endogenous Notch (NICCD) and RBPJ/CB1/Su(H) co-factor binding. Detailed microscopic investigation was performed with Zeiss Imager A1 (in situ hybridizations, Carl Zeiss AG, Jena, Germany) or a Nikon A1 + Laser scanning confocal microscope (in vivo*,* Nikon Corporation, Tokyo, Japan). For detailed microscopic investigations embryos were short time fixed in 4% paraformaldehyde/PBS for 30 min and were mounted in Mowiol. Nuclei were stained by DAPI incubation (1 µg/mL in PBST; 15 min) before mounting. Images were analyzed with ImageJ/Fiji (https://fiji.sc/) and arranged with CorelDraw Graphics Suite (Alludo, Canada) software.

### Plasmid vector cloning and mutagenesis

In vitro HEK 293T transfection experiments were performed with CMV promoter driven human tagged MSGN1 (Origene, Rockville, USA, product-nr. RC225212, CDS: NM_001105569). Patient MSGN1 variant was introduced into the plasmid by site-directed mutagenesis (Q5 Site-Directed Mutagenesis Kit, New England Biolabs/NEB, Ipswich, USA, product-nr. E0554S) and was validated via Sanger sequencing.

Zebrafish injection experiments were performed with *msgn1* promoter driven zebrafish *msgn1* plasmid (sk-tol2-msgn1:mCherry-2A-msgn1) [46]. Human *MSGN1* c.374G > T, p.(Arg125Leu) and zebrafish *msgn1* c.211AG > CT p.(Arg71Leu) missense variants were introduced by site-directed mutagenesis in subcloned coding sequences without start codons (Q5 Site-Directed Mutagenesis and PCR cloning Kit, New England Biolabs/NEB, product-nr. E0554S and E1202S) and were validated via Sanger sequencing. Subsequently, the newly established variant coding sequences were used to replace zebrafish *msgn1* (WT) in sk-tol2-msgn1:mCherry-2A-msgn1 by restriction site cloning. Primer sequences and used plasmids are listed in Additional file 8: Table S2.

### Generation of mutant *msgn1*^*Arg71Leu*^ mRNA and overexpression in zebrafish

A cDNA library was extracted from embryos and wild-type *msgn1* was amplified with PCR using primers listed in Additional file 8: Table S2. Mutant *msgn1* p.(Arg71Leu) was created using overlap extension primers. The wild-type *msgn1* and mutant *msgn1* p.(Arg71Leu) cDNA were cloned into separate pCS2 + vectors between *BamHI* and *EcoRI* restriction sites. In vitro transcription was used to create and isolate mRNA (mMESSAGE mMACHINE™ SP6 Transcription Kit, Invitrogen/Thermo Fisher Scientific, Cat# AM1340). Wild-type embryos were either injected with 200 pg wild-type *msgn1* mRNA (58 embryos) or *msgn1* p.(Arg71Leu) mRNA (54 embryos) at one-cell stage and compared with untreated ones (49 embryos) in three independent experiments. The embryos were fixed at 8-somite stage in 4% paraformaldehyde before in situ hybridization.

### Zebrafish embryo RNA in situ hybridization

RNA in situ hybridization was performed according to standard protocols [39]. RNA probes were synthesized from cloned partial mRNA sequences of target genes using the DIG or FLU RNA Labeling Kit (Roche, Basel, Switzerland, product-nr. 11685619910 and 11175025910). All detected expressions patterns with newly established RNA probes were in accordance with previously published and ZFIN database patterns. Used in situ probes were targeted against: *tbxta* (ZDB-GENE-980526-437; synonyms: *T/ta/brachyury/no tail/ntl*), *msgn1* (ZDB-GENE-030722-1; synonyms: *mespo*), *bmp2a* (ZDB-GENE-980526-388), *tbx6* (ZDB-GENE-020416-5; synonyms: *tbx6r/fss/fused somites/tbx24*), *tbx16* (ZDB-GENE-990615-5; synonyms: *spt/spadetail*). In-situ experiments were performed two times independently and included n ≥ 10 embryos per sample and condition. Detailed information and primers used for probe cloning are listed in Additional file 8: Table S2.

### Image and statistical analysis of RNA in situ hybridization embryos

The embryos were flat mounted via dissection of the yolk sac and imaged under a Nikon SMZ1500 stereomicroscope (HR Plan Apo 1X WD 54), Nikon DS-Ri1 digital camera with reflected light at 23 °C room temperature. FIJI software (ImageJ 1.54f) [32] was used to assess intensity of *tbxta* staining in the tailbud using a standardized circular region (120 µm in diameter, ROI seen in Fig. 4A). Images were first inverted, then the mean intensity of anterior tissue background (17 µm in diameter) was subtracted from the mean intensity of tailbud (ROI). Then each intensity was normalized to the average intensity of uninjected embryos in an entire experiment.

We used unpaired two-tailed Kruskal–Wallis nonparametric test without equal standard deviation assumption in Fig. 4D. The statistical tests and distribution calculations (median and quartiles, confidence intervals) were performed in GraphPad Prism 9.5.0 software.

### Supplementary Information

**Additional file 1**. **Excel file S1:** Raw Data Fig. 3, ISH signal quantification values and injection statistics

**Additional file 2.** **Figure S1: **MSGN1 protein structure computer predictions indicate a conformational change, which can be distinguished between the normal protein structure **(A) **and the p.(Arg125Leu) variant **(B)**. Amino acid alignment of six vertebrate species indicates evolutionary conservation of Arg125 within the basic helix-loop-helix (bHLH) protein domain **(C).** Numbers indicate position within the human amino acid sequence. Uniport amino acid sequence IDs are given for different species

**Additional file 3**. **Figure S2: **Next generation sequencing reads of new MSGN1 missense variant of the affected patient

**Additional file 4**. **Figure S3:** Additional data to *in vitro* transfection of HEK 293T cells with CMV:MSGN1-Tag and CMV:MSGN1-Arg125Leu plasmids at different concentrations presented in Fig. 3. **(A)** Corresponding immunofluorescence controls after 48h of transfection are shown. **(B)** Additional data to quantification of MSGN1 localization in transfected cells. Fluorescence signals in a single z-plane were visualized by confocal laser-scanning microscopy and subsequently signal intensity was measured in single cells (cyto: cytoplasm; nuc: nucleus) and outside of cells (bg: background). Graphs show signal intensity measurements of the DAPI channel (nucleus) of 30 cells per experimental group and 30 background positions. Values are given in Excel file S1

**Additional file 5**. **Figure S4: **Examples of different phenotypes of *msgn1* mosaic zebrafish embryos. Injection with the *msgn1:mCherry-2A-msgn1* plasmid results in different amounts of mCherry positive/*msgn1* expressing cells within the trunk and tailbud region of *tp1:VenusPEST* transgenic embryos. Phenotype severity in the PSM (marked by arrows) 24 h after injection is correlated with *msgn1* overexpressing cell amount and position

**Additional file 6**. **Figure S5: **Different phenotypes of CMV:MSGN1 plasmid injected zebrafish embryos. Injection with the CMV:MSGN1-FLAG-tag (WT) or with the CMV:MSGN1-FLAG-tag p.(Arg125Leu) plasmids, which have been used for *in vitro* cell transfections, results in alteration of tail development also within the trunk and tailbud region of zebrafish embryos at 18 hpf. Injected embryos display axis bending or cell aggregations in the trunk and PSM regions (marked by black arrows). Number of injected embryos are given in Excel file S1.

**Additional file 7**. **Figure S6: **Examples of different phenotypes of *msgn1 p.(Arg71Leu)* and *MSGN1 p.(Arg125Leu)* mosaic *tp1:VenusPEST* zebrafish embryos *in vivo*. Injection with the sk-tol2-msgn1:mCherry-2A-msgn1 p.(Arg71Leu) or with the sk-tol2-msgn1:mCherry-2A-MSGN1 p.(Arg125Leu) plasmid results in mCherry positive/*msgn1* variant expressing cells within the trunk and tailbud region of *tp1:VenusPEST* transgenic embryos. Phenotype severity in the PSM (marked by arrows) in a time frame between 16 and 35 hpf after injection is shown. The observed phenotypes correlate with *msgn1* overexpressing cell amount, position and partly differ in severity between zebrafish and human CDS versions. Number of injected embryos are given in Excel file S1.

**Additional file 8**. **Table S1:** Pathogenicity prediction of *MSGN1* c.374G>T, p.(Arg125Leu) variant. **Table S2:** PCR Primers and DNA plasmids used in this study. **Table S3: **ClinVar missense MSGN1 VUS variants. **Table S4: **ClinVar structural MSGN1 variants.

## Data Availability

The dataset(s) supporting the conclusions of this article is(are) included within the article and its additional files.
