# Supplementary figures and images for: Zebrafish as a model to investigate a biallelic gain-of-function variant in MSGN1, associated with a novel skeletal dysplasia syndrome
Source: Hum Genomics. 2024 Mar 6;18:23. doi: 10.1186/s40246-024-00593-w (PMC10916241; doi:10.1186/s40246-024-00593-w)

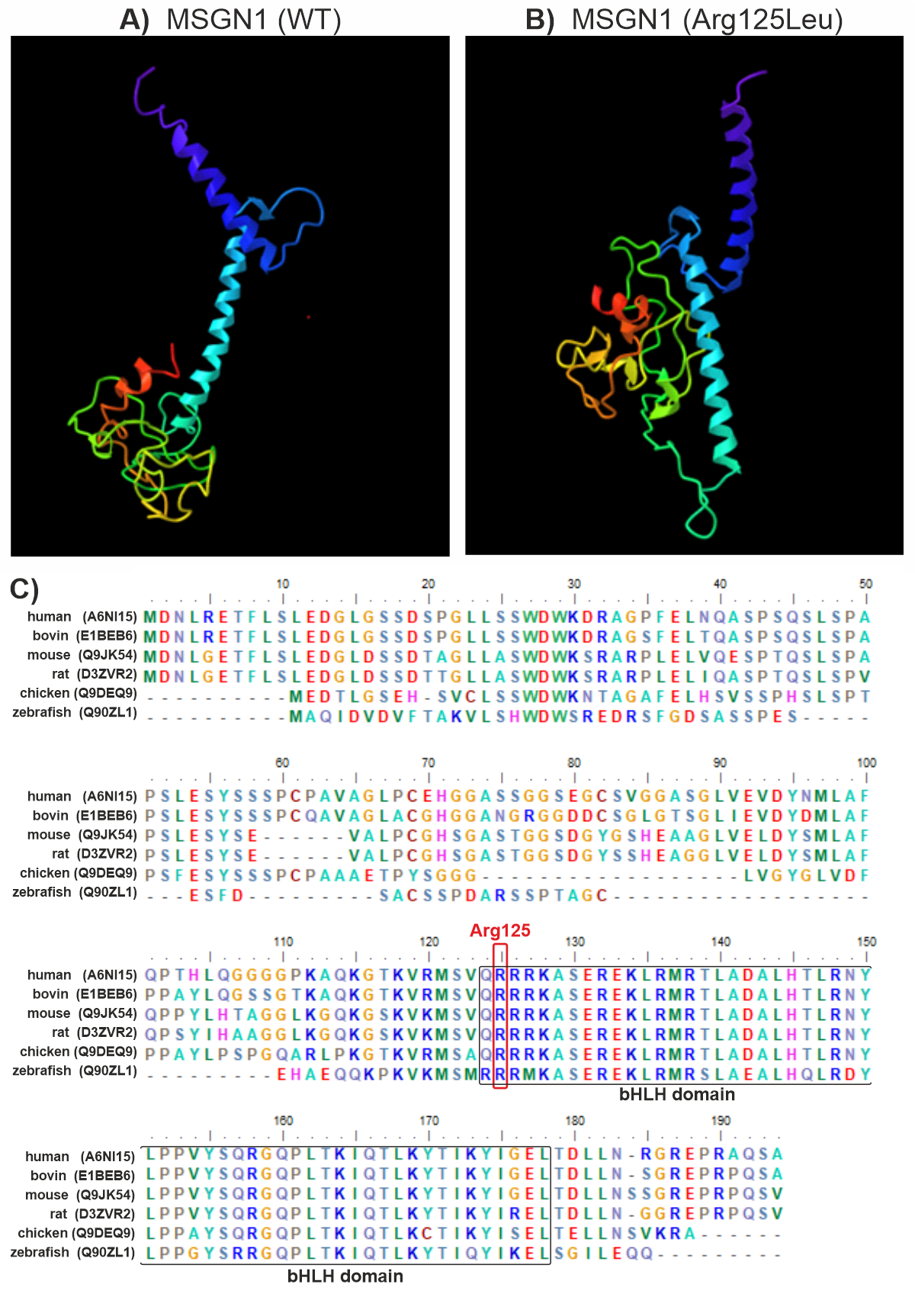

Supplement: Supplementary file 2 — Additional file 2. Figure S1: MSGN1 protein structure computer predictions indicate a conformational change, which can be distinguished between the normal protein structure (A) and the p.(Arg125Leu) variant (B). Amino acid alignment of six vertebrate species indicates evolutionary conservation of Arg125 within the basic helix-loop-helix (bHLH) protein domain (C). Numbers indicate position within the human amino acid sequence. Uniport amino acid sequence IDs are given for different species [file 40246_2024_593_MOESM2_ESM.tif]

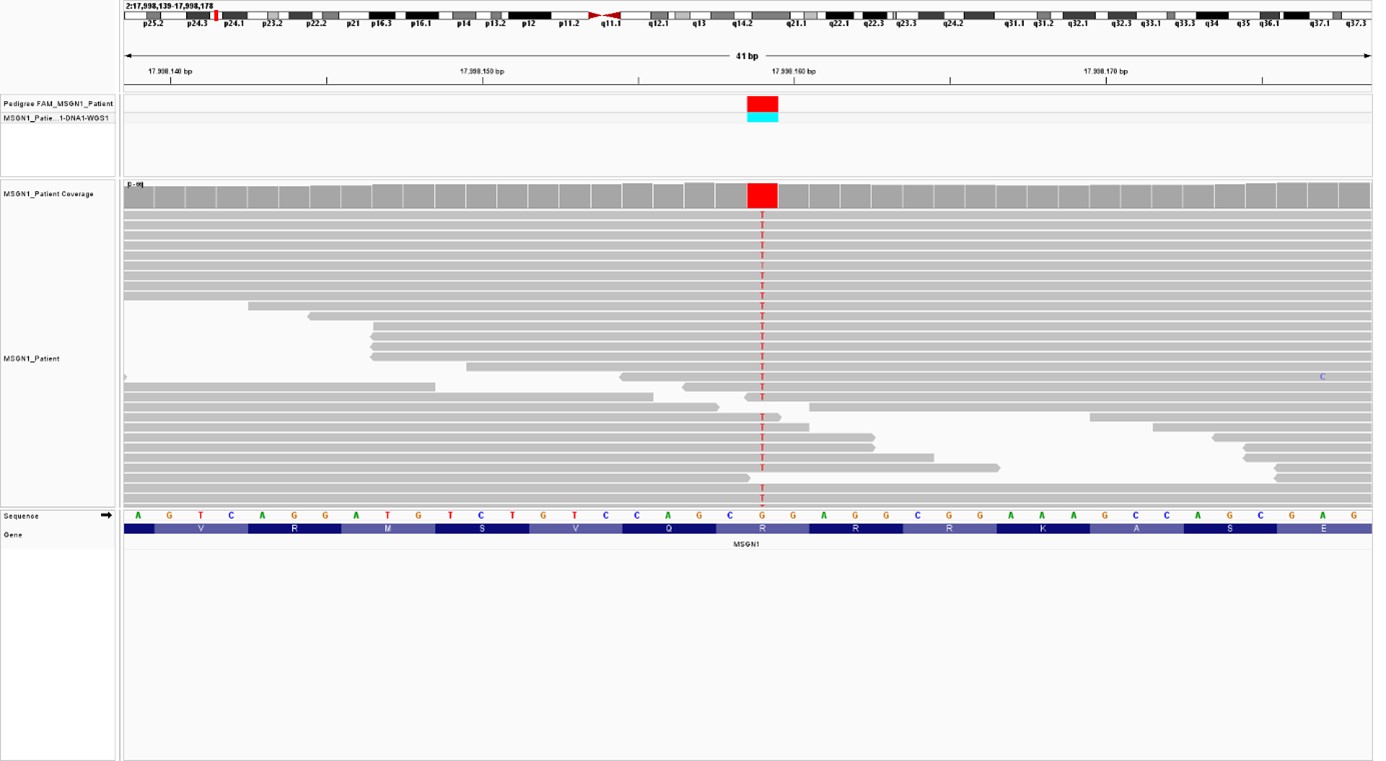

Supplement: Supplementary file 3 — Additional file 3. Figure S2: Next generation sequencing reads of new MSGN1 missense variant of the affected patient [file 40246_2024_593_MOESM3_ESM.jpg]

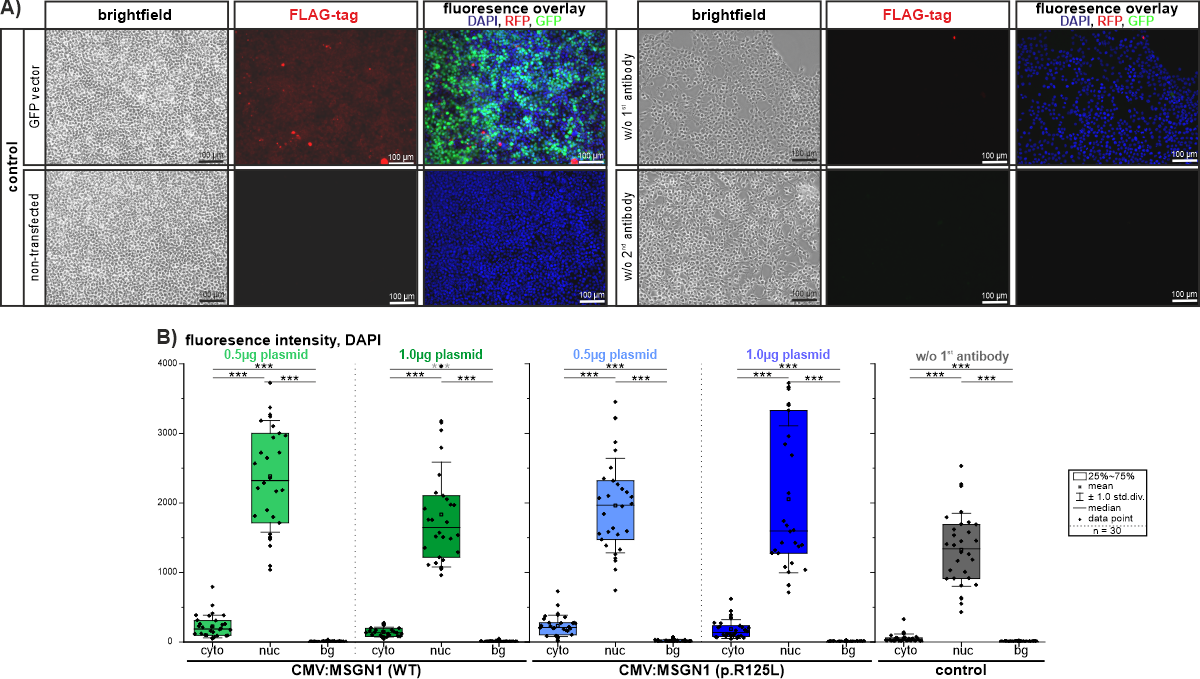

Supplement: Supplementary file 4 — Additional file 4. Figure S3: Additional data to in vitro transfection of HEK 293T cells with CMV:MSGN1-Tag and CMV:MSGN1-Arg125Leu plasmids at different concentrations presented in Fig. 3. (A) Corresponding immunofluorescence controls after 48h of transfection are shown. (B) Additional data to quantification of MSGN1 localization in transfected cells. Fluorescence signals in a single z-plane were visualized by confocal laser-scanning microscopy and subsequently signal intensity was measured in single cells (cyto: cytoplasm; nuc: nucleus) and outside of cells (bg: background). Graphs show signal intensity measurements of the DAPI channel (nucleus) of 30 cells per experimental group and 30 background positions. Values are given in Excel file S1 [file 40246_2024_593_MOESM4_ESM.tif]

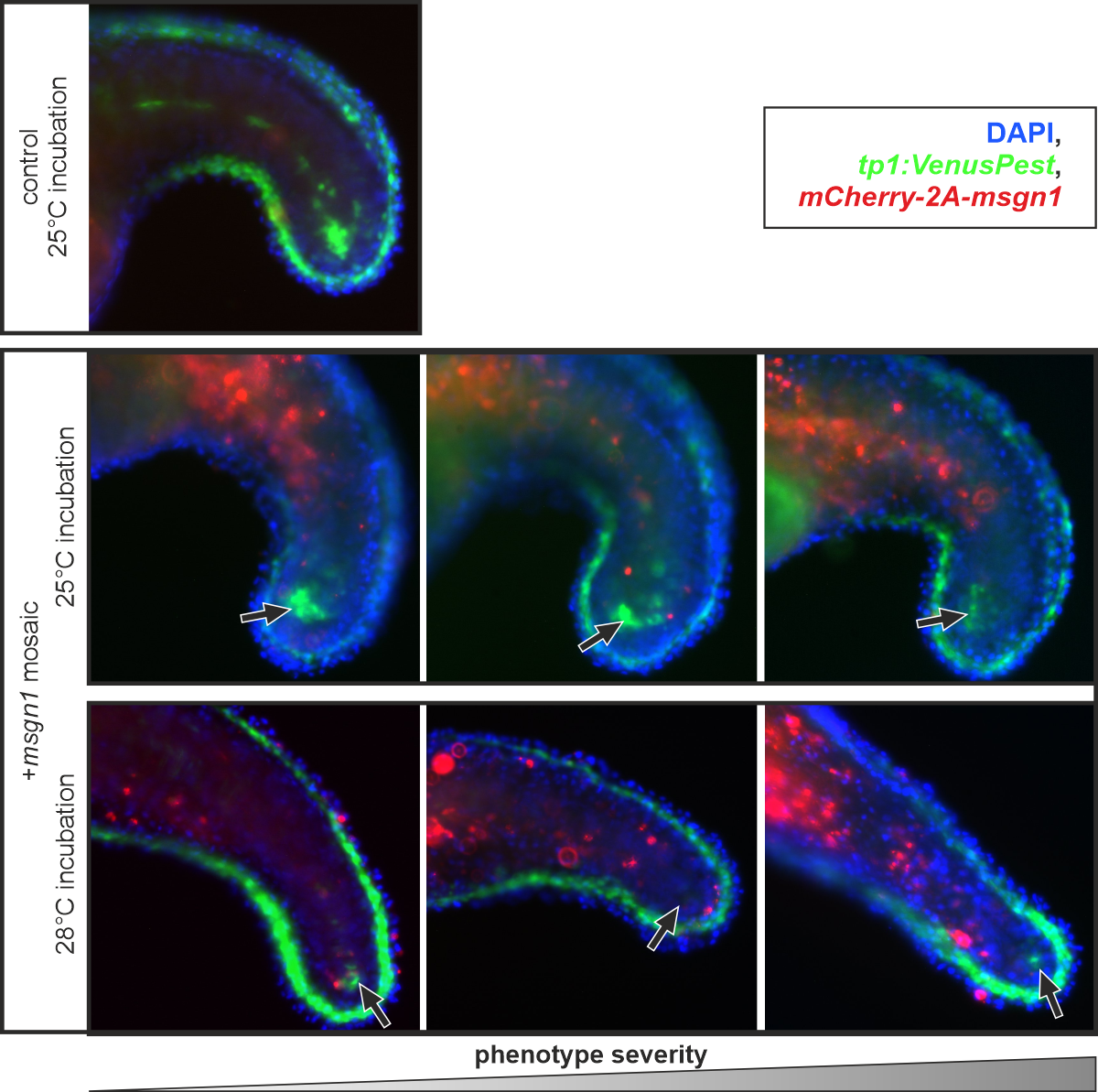

Supplement: Supplementary file 5 — Additional file 5. Figure S4: Examples of different phenotypes of msgn1 mosaic zebrafish embryos. Injection with the msgn1:mCherry-2A-msgn1 plasmid results in different amounts of mCherry positive/msgn1 expressing cells within the trunk and tailbud region of tp1:VenusPEST transgenic embryos. Phenotype severity in the PSM (marked by arrows) 24 h after injection is correlated with msgn1 overexpressing cell amount and position [file 40246_2024_593_MOESM5_ESM.tif]

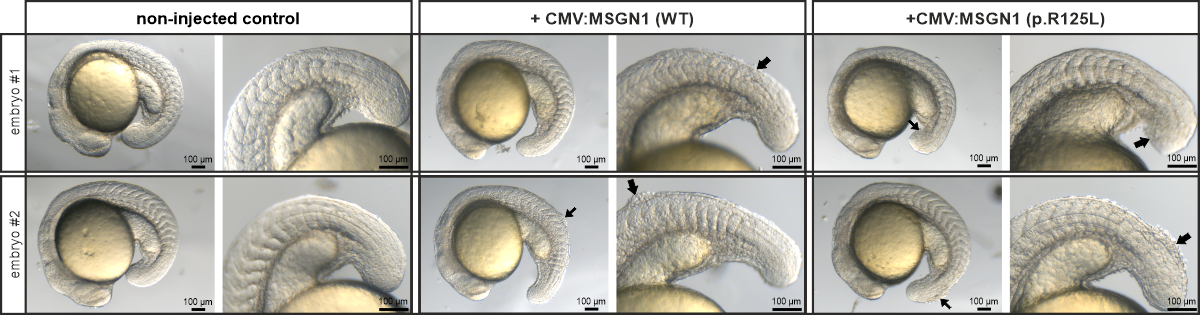

Supplement: Supplementary file 6 — Additional file 6. Figure S5: Different phenotypes of CMV:MSGN1 plasmid injected zebrafish embryos. Injection with the CMV:MSGN1-FLAG-tag (WT) or with the CMV:MSGN1-FLAG-tag p.(Arg125Leu) plasmids, which have been used for in vitro cell transfections, results in alteration of tail development also within the trunk and tailbud region of zebrafish embryos at 18 hpf. Injected embryos display axis bending or cell aggregations in the trunk and PSM regions (marked by black arrows). Number of injected embryos are given in Excel file S1. [file 40246_2024_593_MOESM6_ESM.tif]

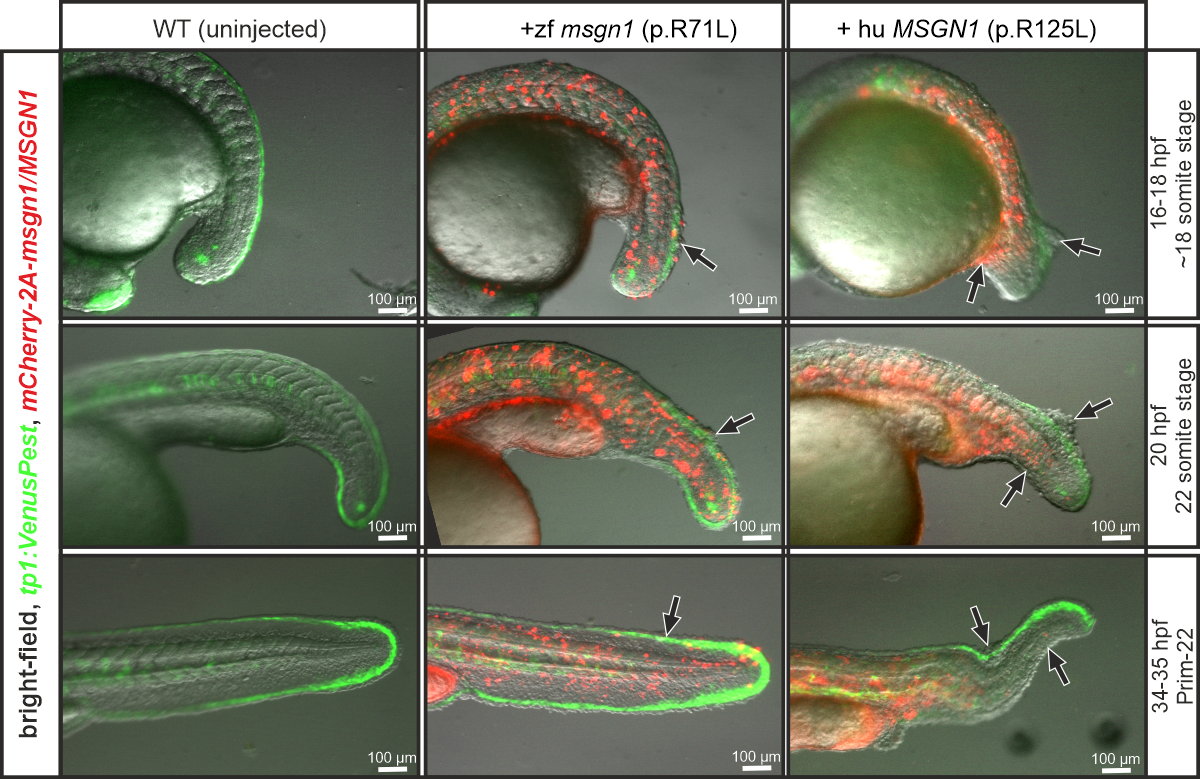

Supplement: Supplementary file 7 — Additional file 7. Figure S6: Examples of different phenotypes of msgn1 p.(Arg71Leu) and MSGN1 p.(Arg125Leu) mosaic tp1:VenusPEST zebrafish embryos in vivo. Injection with the sk-tol2-msgn1:mCherry-2A-msgn1 p.(Arg71Leu) or with the sk-tol2-msgn1:mCherry-2A-MSGN1 p.(Arg125Leu) plasmid results in mCherry positive/msgn1 variant expressing cells within the trunk and tailbud region of tp1:VenusPEST transgenic embryos. Phenotype severity in the PSM (marked by arrows) in a time frame between 16 and 35 hpf after injection is shown. The observed phenotypes correlate with msgn1 overexpressing cell amount, position and partly differ in severity between zebrafish and human CDS versions. Number of injected embryos are given in Excel file S1. [file 40246_2024_593_MOESM7_ESM.tif]
