# Supplementary material for: Zebrafish as a model to investigate a biallelic gain-of-function variant in MSGN1, associated with a novel skeletal dysplasia syndrome
Source: Hum Genomics. 2024 Mar 6;18:23. doi: 10.1186/s40246-024-00593-w (PMC10916241; doi:10.1186/s40246-024-00593-w)
Supplement: Supplementary file 8 — Additional file 8. Table S1: Pathogenicity prediction of MSGN1 c.374G>T, p.(Arg125Leu) variant. Table S2: PCR Primers and DNA plasmids used in this study. Table S3: ClinVar missense MSGN1 VUS variants. Table S4: ClinVar structural MSGN1 variants. [file 40246_2024_593_MOESM8_ESM.docx]

**Table S1:** Pathogenicity prediction of MSGN1 c.374G>T, p.(Arg125Leu) variant.

| IN SILICO TOOLS | PREDICTION |
| --- | --- |
| MetaRNN Score | 0.8713 |
| REVEL Score | 0.77 |
| CADD Score | 31 |
| Alpha Missense | 0.9309 |
| SpliceAI | splice loss: 0.02  splice gain: 0.00 |

**Table S2:** PCR Primers and DNA plasmids used in this study.

| Primer name | Primer sequence (5´-3´) | Amplicon size [bp] | Purpose |
| --- | --- | --- | --- |
| Zf_tbx6_ribo_fwd | CCAAACTCACCAACAACACG | 1045 | *In situ* probe cloning  <http://zfin.org/ZDB-GENE-020416-5> |
| Zf_tbx6_ribo_rev | GCTGAGCTCAAACCTCCATC |  |  |
| Zf_tbx16_ribo_fwd | TGCAAAATCAGCTTGTCTGG | 870 | *In situ* probe cloning <http://zfin.org/ZDB-GENE-990615-5> |
| Zf_tbx16_ribo_rev | CAAGACTCGGGACTCAAAGC |  |  |
| zf_bmp2a_gDNA_fwd1 | TTCGCTAAACAACGCAAGTG | 436 | *In situ* probe cloning  <https://zfin.org/ZDB-GENE-980526-388> |
| zf_bmp2a_gDNA_rev1 | GTCGCTGAAGTCCACGTACA |  |  |
| Hum_MSGN1_forward | GGTGGACTACAATATGTTAGCTTTCC | 189 | MSGN1 exon1 amplification and sequencing  ENSEMBL Transcript: ENST00000281047.4 |
| Hum_MSGN1_reverse | TAGACAGGTGGCAGGTAATTCC |  |  |
| Hum_MSGN1_Muta_fwd | TCTGTCCAGCtGAGGCGGAAA | plasmid | MSGN1 variant mutagenesis |
| Hum_MSGN1_Muta_rev | CATCCTGACTTTGGTGCC |  |  |
| zf_msgn1__mut2Asu _fwd | GAGTATGAGGctGAGGATGAAGG | plasmid | msgn1 variant mutagenesis |
| zf_msgn1__mut2Asu _rev | ATCTTCACTTTCGGCTTC |  |  |
|  |  |  |  |
| Plasmid name | **Source and adaptations** |  | **Purpose** |
| CMV:MSGN1 (WT) | Origene, catalogue #: RC225212 MSGN1 (NM_001105569) Human Tagged ORF Clone, pCMV6-Entry vector |  | *In vitro* transfection,  zebrafish injection |
| CMV:MSGN1 p.(Arg125.Leu) | This study, site directed mutagenesis of CMV:MSGN1 (WT) plasmid |  | *In vitro* transfection,  zebrafish injection |
| pCS2+-msgn1 (WT) | This study,  zebrafish *msgn1* (WT) CDS in pCS2+ plasmid |  | mRNA synthesis |
| pCS2+-msgn1 p.(Arg71Arg) | This study,  zebrafish *msgn1* p.(Arg71Arg) CDS in pCS2+ plasmid |  | mRNA synthesis |
| sk-tol2-msgn1:mCherry-p2A-msgn1 (WT) | Yabe and Takada, 2012 partial zebrafish *msgn1* promoter sequence driving expression of mCherry and zebrafish *msgn1* (WT) CDS |  | zebrafish injection,  *in vivo* investigation |
| sk-tol2-msgn1:mCherry-p2A-msgn1 p.(Arg71Leu) | This study, replacement of zebrafish *msgn1* coding sequence with zebrafish *msgn1* variant in msgn1:mCherry-p2A-msgn1 plasmid |  | zebrafish injection,  *in vivo* investigation |
| sk-tol2-msgn1:mCherry-p2A-MSGN1 p.(Arg125Leu) | This study, replacement of zebrafish *msgn1* coding sequence with human MSGN1 variant CDS in msgn1:mCherry-p2A-msgn1 plasmid |  | zebrafish injection,  *in vivo* investigation |

**Table S3:** Published missense *MSGN1* VUS variants.
 (ClinVar database; https://www.ncbi.nlm.nih.gov/clinvar/; accessed 29.08.2023)

| cDNA Position in NM_001105569.3(MSGN1) | Protein change | Clinical relevance | Accession ID |
| --- | --- | --- | --- |
| c.11T>G p.(Leu4Arg) | L4R | Uncertain | VCV002522663 |
| c.18G>C p.(Glu6Asp) | E6D | Uncertain | VCV002302947 |
| c.82G>T p.(Asp28Tyr) | D28Y | Uncertain | VCV002516087 |
| c.120G>C p.(Gln40His) | Q40H | Uncertain | VCV002354610 |
| c.137G>A p.(Ser46Asn) | S46N | Uncertain | VCV002495774 |
| c.167A>C p.(Tyr56Ser) | Y56S | Uncertain | VCV002263740 |
| c.253G>A p.(Gly85Ser) | G85S | Uncertain | VCV002227906 |
| c.320G>A p.(Gly107Asp) | G107D | Uncertain | VCV002542365 |
| c.352A>G p.(Lys118Glu) | K118E | Uncertain | VCV002204779 |
| c.475G>A p.(Gly159Ser) | G159S | Uncertain | VCV002363247 |
| c.551G>A p.(Arg184His) | R184H | Uncertain | VCV002238447 |

**Table S4:** Published structural interstitial variants encompassing *MSGN1.*
(ClinVar database; https://www.ncbi.nlm.nih.gov/clinvar/; accessed 29.08.2023)
*: variant size <5 Mb; **: variant size >5 Mp (marked in grey); del: deletion; dup: duplication

| Genomic Position, GRCh38/hg38,  variant size and type | Clinical relevance;  number of genes affected | Accession ID |
| --- | --- | --- |
| 2p25.3-23.3  (chr2:12770-25039694)x3 ** ^dup^ | Pathogenic; 655 genes (Last reviewed: Jul 1, 2013); | VCV000153520 |
| 2p25.3-22.3  (chr2:12770-33711509)x3 ** ^dup^ | Pathogenic; 1049 genes (Last reviewed: Jun 18, 2013) | VCV000153441 |
| 2p25.3-24.1  (chr2:17019-20001056)x3 ** ^dup^ | Pathogenic; 500 genes (Last reviewed: Sep 26, 2012) | VCV000152693 |
| 2p25.3-23.3  (chr2:17019-26318846)x3 ** ^dup^ | Pathogenic; 738 genes (Last reviewed: Apr 8, 2011) | VCV000148269 |
| 2p25.3-23.2  (chr2:30341-28419664)x3** ^dup^ | Pathogenic; 895 genes  (Last reviewed: Jul 30, 2009) | VCV000146072 |
| 2p25.3-16.1  (chr2:66097-55570637)x3** ^dup^ | Pathogenic; 1633 genes  (Last reviewed: Aug 12, 2011) | VCV000059133 |
| 2p25.3-21  (chr2:236816-45983232)x3** ^dup^ | Pathogenic; 1402 genes  (Last reviewed: Nov 19, 2013) | VCV000155615 |
| 2p25.3-24.1  (chr2:1664615-23664142)x3** ^dup^ | Pathogenic; 548 genes  (Last reviewed: Nov 4, 2011) | VCV000148848 |
| 2p25.1-11.2 (chr2:7495123-87705899)x3** ^dup^ | Benign; 2459 genes  (Last reviewed: Feb 4, 2013) | VCV000152995 |
| 2p24.2-24.1 (chr2:16723596-1600734)x1*^del^ | Pathogenic; 132 genes  (Last reviewed: Jun 18, 2012) | VCV000150627 |
| 2p25.3-22.3 (chr2:706460-35523639)x3**^dup^ | Pathogenic; 179 genes  (Last reviewed: Apr 15, 2022) | VCV001808629 |
| 2p25.1-q13 (chr2:11504318-111365996)x1**^del^ | Pathogenic; 531 genes | VCV001708190 |
| 2p25.3-q37.3 (chr2:1-243199373)**^dup^ | Pathogenic; complete trisomy chr.2 | VCV001703543 |
| 2p24.3-24.1 (chr2:15640273-19609496)x1*^del^ | Pathogenic; 15 genes  (Last reviewed: Feb 6, 2020) | VCV001180531 |
| 2p24.2 (chr2:17846161-18720906)x3*^dup^ | Uncertain significance; 4 genes  (Last reviewed: Jan 27, 2020) | VCV000979980 |
| 2p24.3-24.1 (chr2:15631145-21729493)x1**^del^ | Pathogenic; 27 genes  (Last reviewed: Jun 29, 2017) | VCV000687179 |
| 2p24.3-24.2 (chr2:12269293-18259781)x3*^dup^ | Pathogenic; 13 genes  (Last reviewed: Jan 26, 2018) | VCV000686578 |
| 2p25.3-q37.3 (chr2:15672-243101834)x3**^dup^ | Pathogenic; 1218 genes  (Last reviewed: Jan 1, 2013) | VCV000616168 |
| 2p25.3-q37.3 (chr2:14238-243048760)x3**^dup^ | Pathogenic; 1218 genes  (Last reviewed: Jan 5, 2017) | VCV000616166 |
| 2p24.2 (chr2:17911847-18217217)x3*^dup^ | Benign; 4 genes  (Last reviewed: Jul 14, 2016) | VCV000603701 |
| 2p24.2 (chr2:16926193-18225507)x3*^dup^ | Likely benign; 6 genes  (Last reviewed: Jan 21, 2020) | VCV000562638 |
| 2p24.2 (chr2:17814030-18851257)x3*^dup^ | Uncertain significance; 8 genes  (Last reviewed: Jun 23, 2017) | VCV000446356 |
| 2p25.3-24.1 (chr2:12770-20081474)x3**^dup^ | Pathogenic; 67 genes  (Last reviewed: Aug 15, 2014) | VCV000443675 |
| 2p25.3-q37.3 (chr2:12771-242783384)** ^dup^ | Pathogenic; 1216 genes  (Last reviewed: Jul 14, 2015) | VCV000442998 |
| 2p25.3-q37.3 (chr2:12771-242783384)x3**^dup^ | Pathogenic; 1216 gens  (Last reviewed: Dec 2, 2014) | VCV000442997 |
